# Effects of Long-Term Wear and Discontinuation of Orthokeratology Lenses on the Eyeball Parameters in Children with Myopia

**DOI:** 10.7150/ijms.79496

**Published:** 2023-01-01

**Authors:** Qin Zhu, Jie Yin, Xuejiao Li, Min Hu, Liping Xue, Jieying Zhang, Yuan Zhou, Xiaofan Zhang, Yingting Zhu, Hua Zhong

**Affiliations:** 1 Department of Ophthalmology, the First Affiliated Hospital of Kunming Medical University, Kunming 650031, China.; 2 Department of Pediatric Ophthalmology, Affiliated Hospital of Yunnan University; Kunming 650021, China.; 3 BioTissue (Tissue Tech, Inc.), Ocular Surface Center, and Ocular Surface Research & Education Foundation, Miami, FL, 33126 USA.

**Keywords:** myopia, children, orthokeratology, axial length, central corneal thickness, anterior chamber depth, central lens thickness

## Abstract

**Objective:** To evaluate the effects of long-term wear and discontinuation of the orthokeratology lenses (Orth-K) on the biological parameters of eyeballs in children with myopia.

**Methods:** In this prospective study, a total of 308 subjects with myopia were randomized to receive Orth-K (n = 154) or single vision spectacles (SVS) (n = 154) for 12 months followed by a 1-month withdrawal period. The axial length (AL), the central corneal thickness (CCT), the anterior chamber depth (ACD) and the central lens thickness (CLT) were assessed at the baseline, 6 months, 12 months, and 13 months (1-month after lens withdrawal).

**Results:** A total of 279 subjects completed the 13-month follow-up (142 in Orth-K group and 137 in SVS group). No statistical difference was noted in AL, CCT, ACD and CLT between the two groups at the baseline (all p > 0.05). However, compared with the baseline, the AL from the two groups became elongated 12 months after wearing Orth-K or SVS. The increase of AL in Orth-K group was 0.22 ± 0.11 mm, significantly smaller than 0.35 ± 0.08 mm in SVS group (p < 0.05). In addition, CCT in Orth-K group was 544.26 ± 11.69 µm at 12 months, significantly thinner than 550.49 ± 12.13 µm in SVS group (p < 0.05). Interestingly, the change in CCT between the baseline and 1-month after withdrawal of the lens was not statistically different in either group (all p > 0.05). Furthermore, at 12-months, CLT in Orth-K group was 3.35 ± 0.21 mm, significantly thicker than 3.31 ± 0.15 mm at baseline and thicker than 3.30 ± 0.05 mm in SVS group at 12 months (all p < 0.05). Lastly, ACD was not statistically different between Orth-K and SVS groups at any time point (p > 0.05).

**Conclusion:** Orthokeratology lenses can effectively retard axial elongation, reversibly reduce CCT, increase CLT in myopic children, but have no obvious effect on ACD, indicating that Orth-K may significantly retard myopia without noticeable myopia rebound after interruption of Orth-K.

## Introduction

Myopia is increasingly affecting public health due to its increasing incidence and high public health costs. In the past 20 years, the overall prevalence of myopia worldwide has increased from 79.5% to 87.7%, including moderate myopia (38.8% to 45.7%), severe myopia (7.9% to 16.6%) and high myopia (0.1% to 0.9%) 1. By 2050, nearly 50% of the population may have myopia 2. In China, the prevalence of myopia in primary, middle, high schools and universities is 27.0%, 81.0%, and 78.5%-95.5% respectively 3, 4. Complications associated with high myopia, such as retinal detachment, macular hole and macular degeneration, are important causes of blindness, and the risk of these complications increases with the severity of myopia. Unfortunately, the mechanism for the progression of myopia is still unknown. Nevertheless, several theories have been developed to explain the etiology of myopia, including too few outdoor activities, too long near-vision work and accelerated urbanization 5-7. These theories have suggested that increasing outdoor activities and reducing near-vision work may help delay the progression of myopia 8, 9. Clinically, orthokeratology lenses (Orth-K) and atropine eye drops are currently in use for retarding juvenile myopia progression 10-12 (also reviewed in 13).

Atropine is considered a concentration-dependent M receptor blocker. Due to dilation and ciliary muscle paralysis by atropine, the patients may have severe and persistent photophobia and near-ambiguity. In addition, significant refractive regression may develop after withdrawal of high-dose atropine 14-16. Although lower concentrations of atropine have mild side effects 17-19, it is less effective and have not been approved by the State Drug Administration in China. In addition, the potential crystal and retinal photo damage of atropine has become a major concern for its use 20. In contrast, Orth-K lens were made by a rigid gas permeable contact lens material 21, which can reshape the corneal morphology and temporarily reduce the curvature of the central region of the cornea, increase the curvature of the peripheral region, and form a myopic defocus on the peripheral retina. Myopic defocus has been shown to significantly delay the progression of myopia 22 and to be safe and effective in adolescent patients 23-25. Therefore, Orth-K lens treatments provide an alternative and effective method for the treatment of myopic progression, which may reduce axial elongation by 55% after 24 months 21.

In this study, myopia adolescents were selected to explore the effect of Ortho-K lens on eyeball parameters using the traditional single-vision spectacles as the control, which indicates that Ortho-K lens can effectively control myopia progression through inhibition of axial elongation. In addition, Orth-K lens can also reversibly decrease CCT and increase of CLT without effect on ACD. Such a controlled study may provide useful information for effective control of myopia in adolescents worldwide.

## Materials and methods

### General information

A prospective study was conducted in 308 myopia adolescents from December 2019 to December 2021, with an average age of (9.20 ± 1.51) years from the Department of Ophthalmology, the First Affiliated Hospital of Kunming Medical University and the Department of Pediatric Ophthalmology, the Affiliated Hospital of Yunnan University, China. The patients were randomly divided into Orth-K group (154 patients with Orth-K), and SVS group (154 patients with SVS). All myopia children and their legal guardians were given informed consent containing the purpose and the significance and the eye examination process of this study. This study followed the Helsinki Declaration and was approved by the Ethics Committee of the Affiliated Hospital of Yunnan University, China.

### Subject inclusion and exclusion criteria

Inclusion criteria: (1) diopter -5.0 D to -1.00 D (cycloplegia), conforming corneal astigmatism less than 1.50 D, Inverse corneal astigmatism less than 0.75 D; (2) corneal topography, the minimum curvature of the cornea (D) minus the expected reduction greater than 36 D, (3) corneal diameter of 10.9 - 12.5 mm, best-corrected visual acuity (BCVA) > 0.0 logMAR; (4) myopia children, 8 - 12 years old, willing to participate in 13-month observation.

Exclusion criteria: (1) with organic diseases of the eye, such as strabismus, congenital cataract and optic nerve dysplasia; (2) with a history of ocular surgery; (3) suffering from visual functional systemic diseases such as diabetes and chromosomal abnormalities; (4) with previous use of contact lenses, bifocal or multifocal lenses, or other myopia treatments (such as atropine eye drops); (5) patients with intraocular pressure higher than 21 mmHg; (6) unable to understand the mechanism of action of Orth-K and potential problems and limitations of patients with correction; (7) patients with disturbance of consciousness or unconsciousness.

### Methods

After systematic ocular examinations, the corneal curvature, the corneal and pupil diameters were measured by the corneal topography (Medmont E300, Australia). The axial length (AL), the central corneal thickness (CCT), the anterior chamber depth (ACD) and the central lens thickness (CLT) were examined using IOL Master 700 (Carl Zeiss 700, Germany). The refractive error in each eyeball was subsequently measured by the autorefractor (KR-8800, Topcon, Tokyo, Japan) after sufficient cycloplegia cyclopentanone eye drops (1%), and three designed measurements were averaged. The refractive error was recorded as Sphere (S), Cylinder (C) and Axis (A), and then the spherical equivalent refraction (SER) was calculated using the sum of the spherical power and half of the cylindrical power (SER=S+C/2). The slit lamp (SL-D8Z, Topcon Optical, Tokyo, Japan) was used to check the anterior segment of the eye before and after wearing the orthokeratology lens and to evaluate the suitability of the lens. The participators in SVS group were given SVS for wearing, while the children in Orth-K group were given Orth-K for wearing. The keratoplasty parameters included: lens material, Boston XO; lens diameter, 9.80 - 11.60 mm; optical diameter, 5.50 - 6.00 mm; oxygen permeability coefficient, 100 × 10 ^-11^ (cm^2^ / s). After wearing Orth-K or SVS, the patients were re-examined regularly. The patients in Orth-K group returned to the hospital the next day, 1 week, 1 month, and then once every 3 months, and the patients in SVS group returned to the hospital every 3 months. Uncorrected visual acuity (UCVA), SER, BCVA, AL, CCT, ACD and CLT were examined at each follow-up. In addition, the safety and health of the cornea was monitored by fluorescein staining under the ophthalmological slit lamp microscope at each follow-up visit. In case of abnormalities such as corneal punctate epithelial shedding, artificial tears were administered on an as-needed basis. When the corneal epithelium was obviously damaged, or when the child had systemic diseases such as cold and fever, the wearing was suspended to ensure safety. All the observation intervals and the examinations for the children in SVS group were the same as those in Orth-K group.

### Observations

To compare the biological parameters of the eyeballs before and after wearing lens, cycloplegia was performed for the patients using cyclopentanone eye drops (1%) by examining whether the light reflection still existed. It was necessary to add eye drops again until the light reflection disappeared. Then the data were measured and recorded. BCVA, AL, SER, CCT, ACD and CLT were measured and recorded before wearing glasses, after wearing glasses for 6 months and 12 months, and after withdrawal for 1 month.

### Sample size

Based on previous findings, we anticipated a mean (SD) AL progression of 0.3 (0.4) mm by SVS in the control group throughout a 12-month period. We calculated 50% reduction in AL progression (reducing axial elongation by 0.15 mm) by Orth-K for the treatment group, compared with that from the control group. The sample size required was 123 participants per group, or a total sample size of 246 participants for an α level of 0.05 (2-tailed), a power of 90%. Adjusting for 20% loss to follow-up, a total sample size of the participants was equal to 308.

### Statistics

SPSS version 26.0 software was used for statistical analysis. The counting data were analyzed by χ2 chi-square test. The data were expressed as mean ± standard deviation. The data between SVS and Orth-K groups were analyzed by analysis of variance (ANOVA) and independent t-test. p < 0.05 was considered as statistically significant.

## Results

### The baseline data

308 eligible schoolchildren were recruited for this investigation and randomly allocated to Orth-K group (n=154) and SVS group groups (n=154). A total of 279 subjects completed the 13-month follow-up (142 in Orth-K group and 137 in SVS group).

No statistical difference in age, gender, equivalent spherical degree, and AL between Orth-K and SVS groups before treatment was noted (p > 0.05, Table 1).

### The axial length (AL)

No statistical difference in AL between the two groups was noted before wearing glasses (all p > 0.05, Table 2). After 6 months and 12 months, the AL in both treatment groups increased. However, the AL in the Orth-K group was significantly smaller than that in SVS group in 6 months, 12 months and 1 month after withdrawal (all p < 0.01, Table 2). Furthermore, the change in AL from the baseline to 12 months in the Orth-K group (0.22 ± 0.11 mm) was significantly less than that of SVS group (0.35 ± 0.08 mm) (p < 0.001). After withdrawal of Orth-K for 1 month, AL remained relatively stable, with no significant increase statistically when compared with that before withdrawal.

### The central corneal thickness (CCT)

No statistical difference was noted between Orth-K and SVS groups before wearing glasses (p > 0.05). After 6 months of wearing glasses, the CCT in the Ortho-K group was significantly thinner than the baseline value (p < 0.001, Table 3). After 12 months of wearing glasses, the difference between CCT change in Orth-K group (7.32 ± 1.21 µm) and in SVS group (1.05 ± 0.18 µm) was statistically significant (p < 0.001). At 1 month after withdrawal of Orth-K, CCT recovered to the baseline level (p > 0.05).

### The central anterior chamber depth (ACD)

No statistical difference was noted between the two groups for ACD (p > 0.05) before wearing glasses. After wearing glasses for 6 and 12 months and after its withdrawal for 1 month, no significant changes were noted in the depth of the anterior chamber (all p > 0.05, Table 4).

### The central lens thickness (CLT)

No statistical difference was noted between SVS and Orth-K groups for CLT before wearing glasses (p > 0.05). After wearing glasses for 6 and 12 months and after its withdrawal for 1 month, CLT from Orth-K group increased significantly when compared to the SVS group (all p < 0.01). In particular, after 12 months of wearing glasses, the difference between Orth-K and SVS groups (0.04 ± 0.02 mm and -0.01 ± 0.02 mm respectively) was statistically significant (p < 0.05). After withdrawal for 1 month, CLT from Orth-K group did not change significantly when compared to that before withdrawal (Table 5).

**Figure 1:**
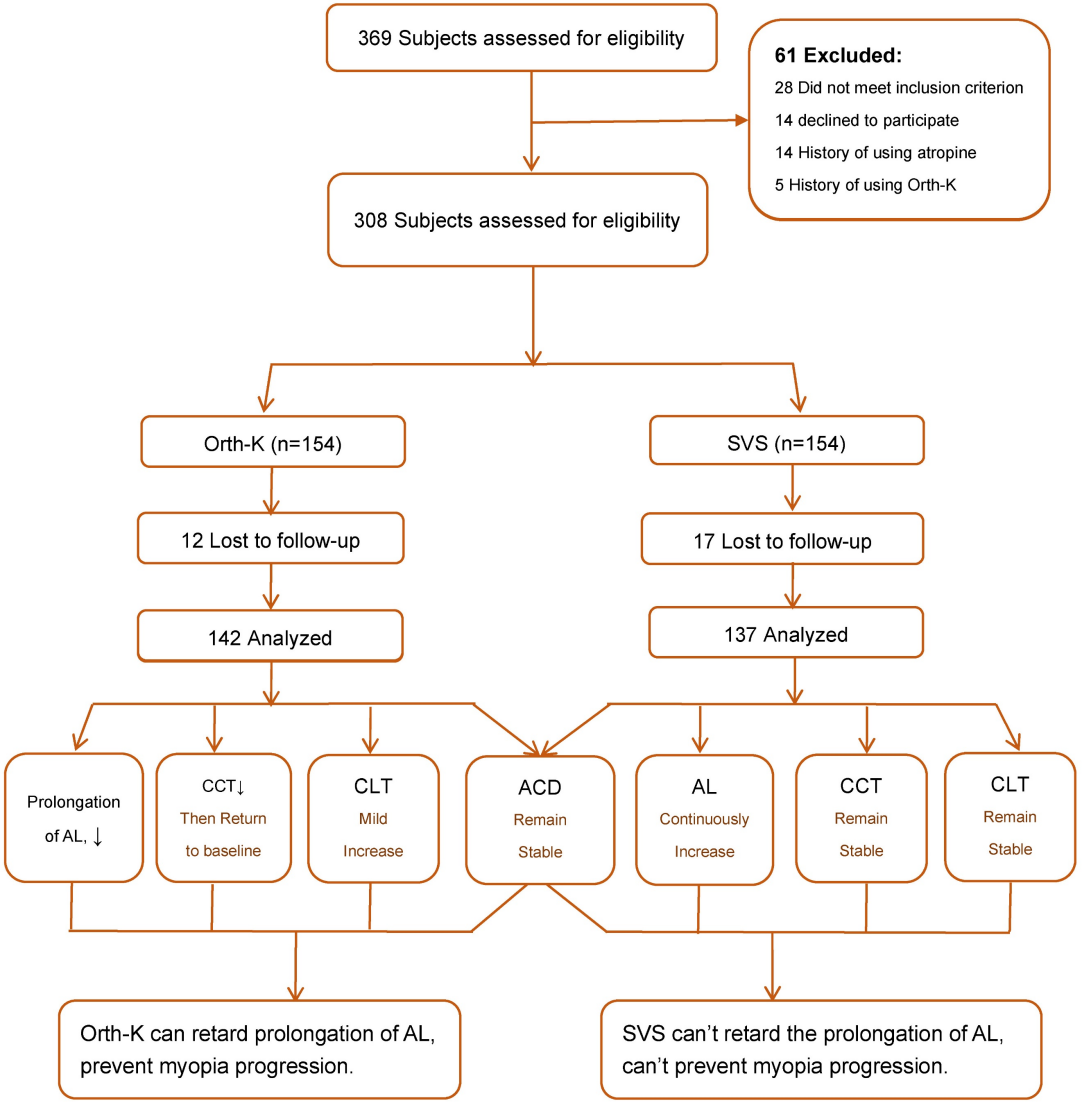
Flow chart of subject recruitment and research procedure.

Summary, the results can be summarized as Figure 1.

## Discussion

The clinical incidence of myopia in adolescents is high, and it has become an important public health problem worldwide. At present, the number of myopia patients in China is increasing steadily. In fact, the incidence of myopia in 6-year-old children is 14.5%, but increases to 36.0% in primary school students, 71.6% in junior high school students, and 81.0% in senior high school students 26, 27, suggesting that myopia is in a progressive trait, causing serious damage to patients' vision and increasing burden to families and societies. Therefore, how to effectively prevent the onset of myopia and control the progression of myopia has become critical issues in this world. As an example, orthokeratology has been widely used for treatment of myopia in adolescents. The material used for the orthokeratology is a rigid gas permeable material. It adopts a special reverse geometry design to reshape corneal morphology, temporarily reducing the curvature of the central region of the cornea, increasing the curvature of the peripheral corneal region, and forming a myopic defocusing of the peripheral retina 28. As an external object placed against the front surface of the cornea, orthokeratology lens is expected to cause certain changes in the anterior segment parameters, such as corneal curvature and thickness 29. In this study, the biological parameters of eyeballs with myopia were evaluated after wearing Orth-K or spectacles to determine their role in delaying myopia progression.

Retardation of AL is an important indicator for controlling the progression of myopia 30. Previously, some researchers performed orthokeratology and spectacles treatment in Chinese children aged 7-13 years with myopia 31. During follow-up, the patients showed AL prolongation, nevertheless, the morphological changes to orthokeratology became smaller, which is consistent with our study. In this study, we report that after wearing Orth-K for 6 and 12 months, AL prolongation in the Orth-K group was slowed by 0.13 mm compared with that from SVS group, suggesting that Orth-K can prevent AL prolongation. Interestingly, no significant rebound in AL after Orth-K withdrawal is noted, indicating that no significant refractive retraction occurs after the orthokeratology lens is removed.

After wearing Orth-K, the corneal epithelial tissue is reconstructed, leading to corneal remodeling. A study has reported that the effect of Orth-K on CCT and morphology is reversible 32. However, the concept is still controversial. In this study, anterior segment OCT was used to observe the changes of CCT in adolescents after wearing Orth-K. Our results show that CCT in Orth-K group was thinner than that of SVS group [(544.26 ± 11.69) vs (550.49 ± 12.13) µm] at 12 months (Table 3). However, after withdrawal of Orth-K for 1 month, CCT increased to 551.14 ± 12.26 µm (Table 3), which is relatively the same as the baseline level. This indicates that after withdrawal of Orth-K, CCT of the patients can be restored to the original state (Table 3). Interestingly, CCT returned to normal 1 month after keratoplasty (Table 3), indicating that long-term wear of Orth-K has no significant effect on central corneal and peripheral thickness.

The anterior chamber is an important part of the human eye affecting the aqueous humor circulation, which maintains stability of intraocular pressure. Observing the influence of Orth-K on the anterior chamber has a very significant clinical significance 33. In this study, our results show that the ACD in the Orth-K group is 3.30 ± 0.08 mm at 6 months, 3.30 ± 0.07 mm at 12 months, and 3.31 ± 0.09 mm at 1 month after withdrawal (Table 4). This suggests AL remains stable, with no significant difference (p>0.05) compared with ACD before wearing Orth-K (3.30 ± 0.12 mm) (Table 4). After wearing Orth-K and SVS for 6 and 12 months, no significant difference was noted in the change in the depth of the anterior chamber (Table 4), suggesting that Orth-K can mainly correct the myopia by changing curvature of the anterior surface of the cornea, and has no significant effect on the posterior surface of the cornea and the depth of the anterior chamber. Due to the compression of the cornea in the base arc region, the central corneal epithelium and shallow stromal layer are flattened rather than curved throughout the cornea. The stable posterior surface of the cornea and the depth of the anterior chamber improve the predictability of Orth-K. It is only necessary to consider how to change the shape of the anterior surface of the cornea during lens design.

Previous studies have found that CLT is basically stable after 10 years of age, and CLT may be altered in patients with ametropia 34, in which CLT may be thin due to decreased lens accommodation and other factors 35. Our results show that CLT is slightly thinned after wearing SVS (Table 5). This may be due to the relative farsightedness of the peripheral retina, causing AL elongation. Changes in AL can further cause compensatory changes in CLT 36.

### Limitations

This study has some limitations. Firstly, the study was not double blinded. However, the outcomes were generated on objective measurements by experienced professionals, likely minimizing this observation bias. Secondly, the duration of study was 12 months, not a very long term for a clinical trial. Nevertheless, the results were reliable and significant. A longer-term trial has been planned in our group. Thirdly, this study did not include a long-term withdrawal period for children who continued to use Orth-K for 12 months. Therefore, further investigation is needed to confirm the rebound effect at a longer term. Fourthly, anxiety among Chinese parents, unwillingness to join the investigation, and uncooperative patient attitude were other factors affecting this study. However, this effect was very limited since not many children were withdrawn during this study due to our extraordinary effects to complete this investigation.

## Conclusions

In summary, for patients with myopia in primary and secondary schools, wearing Orth-K can effectively inhibit the axial elongation, reversibly reduce CCT, and increase CLT. However, it has no significant effect on ACD (summarized in Figure 1). Therefore, we conclude that Orth-K can effectively delay the progression of myopia in adolescents, without significant refractive regression after withdrawal of Orth-K. Such a clinical application method can be recommended for effective control of myopia progression.

## Figures and Tables

**Table 1:** Statistical characteristics of the baseline data.

	Orth-K Group (n =142)	SVS Group (n =137)	p Value
Male/Female	70 / 72	68 / 69	0.16
Age	9.18 ± 1.29	9.23 ± 1.68	0.12
Initial SER (D)	-2.74 ± 0.39	-2.73 ± 0.41	0.29
AL (mm)	23.53 ± 0.19	23.40 ± 0.16	0.28

**Table 2:** Comparison of AL levels (mm) before and after wearing glasses (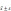)

Group	Before Wearing	6 Months after	12 Months after	1 Month after Withdrawal	F	P
Orth-K	23.18 ± 0.66	23.22 ± 0.68	23.40 ± 0.68	23.40 ± 0.69	16.67	0.000
SVS	23.17± 0.65	23.39 ± 0.69	23.52 ± 0.70	23.52 ± 0.71	20.59	0.000
t	0.711	2.736	0.629	0.821	--	--
p	0.484	0.006	<0.001	<0.001	--	--

**Table 3:** Comparison of CCT levels (µm) of eyeballs before and after wearing glasses (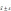)

Group	Before Wearing	6 Month after	12 Months after	1 Month after Withdrawal	F	p
Orth-K	551.58 ± 11.32	544.34 ± 13.37	544.26 ± 11.69	551.14 ± 12.26	12.780	0.00
SVS	551.54 ± 11.39	551.36 ± 12.23	550.49 ± 12.13	551.17 ± 12.12	16.976	0.000
t	0.185	4.678	5.189	6.213	--	--
p	0.849	0.000	0.000	0.569	--	--

**Table 4:** Comparison of ACD levels (mm) in the eyeball before and after wearing glasses (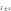)

Group	Before Wearing	6 Months after	12 Months after	1 Month after Withdrawal	F	p
Orth-K	3.30 ± 0.12	3.30 ± 0.08	3.30 ± 0.07	3.30 ± 0.10	3.153	0.190
SVS	3.31 ± 0.11	3.31 ± 0.08	3.31 ± 0.07	3.31 ± 0.10	2.312	0.213
t	0.540	7.567	1.001	0.814	--	--
p	0.568	0.181	0.237	0.207	--	--

**Table 5:** Comparison of CLT levels (mm) in the eyeball before and after wearing glasses (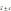)

Group	Before Wearing	6 Month after	12 Months after	1 Month after Withdrawal	F	p
Orth-K	3.31 ± 0.15	3.33 ± 0.14	3.35 ± 0.21	3.35 ± 0.31	12.190	0.012
SVS	3.31 ± 0.07	3.31 ± 0.05	3.30 ± 0.23	3.31 ± 0.32	13.436	0.000
t	0.567	7.421	7.279	6.239	--	--
p	0.561	0.007	0.006	0.007	--	--
